# The protective effect of *Costus afer* Ker Gawl aqueous leaf extract on lead-induced reproductive changes in male albino Wistar rats

**DOI:** 10.5935/1518-0557.20190019

**Published:** 2019

**Authors:** Anthonet Ndidiamaka Ezejiofor, Orish Ebere Orisakwe

**Affiliations:** 1

**Keywords:** sperm analysis, lead acetate, Costus afer, reproductive damages

## Abstract

**Introduction:**

Lead is a multiple organ toxicant and an oxidative-stress inducer. The effect of *Costus afer* on metal- induced male reprotoxicity has not been previously carried out, hence this study. The present study investigates the protective effect of *Costus afer* aqueous leave extract on lead- induced reproductive damages in male albino Wistar rats.

**Methods:**

Adult male albino Wistar rats were weighed and separated into five groups of five rats each. Groups 1 & 2 served as normal and toxic controls receiving deionized and leaded (CH_3_COO)_2_Pb.3H_2_O and water respectively. Groups 3, 4 and 5 were given 750, 1500 and 2250mg/kg of *Costus afer* orally, respectively while receiving Pb^2+^ water *ad libitum* for 28 days.

**Results:**

The reproductive and antioxidant parameters obtained from the result served as scientific evidence in the study. The result showed non-significant changes in the absolute and relative weights of epididymis and testes in the Pb Group *versus* the control. Significant increases were recorded in the sperm analysis, blood lead (7.9±1.02; 1.1±0.01) level (BLL), luteinizing hormone (LH) (8.5±1.4:5.5±0.4), and a decrease in follicle stimulating hormone (FSH) (4.5±2.6:6.5±1.65), with non-significant changes in testosterone (TET) (1.3±0.00:1.6±0.2) in the Pb group compared to the control.

**Conclusion:**

The treatment with *Costus afer* exhibited dose-dependent significant changes in testicular oxidative stress, hormonal, sperm analysis and histopathological changes induced by lead. Aqueous leaves extract of *Costus afer* may be protective against lead induced testicular damage.

## INTRODUCTION

Plants represent a major part of the therapeutic ingredients in almost all systems of medical science. Herbal therapy in Africa is an age-long practice. Men’s continuous reliance on herbs for therapeutic and nutritional benefits cannot be overemphasized. Herbs and herbal products, or herbal supplements are all forms of plant materials used as complementary or alternative medicines throughout the world. *Costus afer* (bush cane, ginger lily) is a widespread tropical plant commonly found in shabby forest and riverbanks of West Africa (Iwu, 1993).

The study carried out by previous researchers (Morán-Martínez *et al*., 2013), reported the role of lead in male reproductive toxicity and its implication in infertility. Led buildup in the testis is known to have anti-spermatogenic effects, as per reported by Fahim *et al.* (2013). According to Anjum *et al.* (2017), the testis of lead-treated rats revealed remarkable degeneration and atrophied seminiferous tubules, with absence of regular differentiated stages of germ cells to mature spermatozoa. Given the high cost, scarcity and wide range of adverse effects of chelators, such as the classical antidotes of lead poisoning, continuous search for widely available ‘’natural antidotes’’ that will ameliorate or reverse the deleterious effects of lead in developing nations has been the research focus in our laboratory. The present study seeks to examine the efficacy of *Costus afer* in mitigating lead-induced oxidative stress and injury in the male reproductive system of male albino Wistar rats.

## MATERIALS AND METHODS

### Plant identification

The plant was identified and authenticated by Mr O.Ozioko A.O, International Center for Ethno Medicine and Drug Development (INTERCEDD), University of Nigeria Nsukka, Nigeria and the voucher Number is INTERCEDD/033.

### Sample processing and extraction

The leaves were washed with clean water, shade-dried in a well-ventilated place for 24hrs. Two-hundred and fifty grams of the leaves were weighed and macerated into 500ml of deionized water, placed in a closed container and allowed to stand for 48hrs, under constant stirring. After 48hrs, the mixture was strained, the marc pressed, and the liquid filtered and stored in a refrigerator at 4ºC. The solution was discarded every three days and a fresh sample prepared, and the process was repeated until the end of the study.

### Preparation of 2500-ppm leaded-water

A 50g of lead acetate (CH_3_COO)_2_Pb·3H_2_O were dissolved in 12ml of 1N HCl and made up to 20L with deionized water. Ten grams of glucose was added to improve the taste according to Sadeghi *et al.* (2013).

### Animal Husbandry

Twenty male albino Wistar rats (*Rattus norvegicus*) weighing between 90-180g were purchased from the Department of Experimental Pharmacology & Toxicology - Animal house Abuja campus, Faculty of Pharmaceutical Sciences, University of Port-Harcourt, Rivers State. The rats were kept in polypropylene cages and maintained under the standard conditions prescribed by the committee for the purpose of controlling and supervising animal experiments (CPCSEA). The Institutional Animal Ethics Committee under the following approval number approved the experimental protocol: UPH/PHARM/2017/033. They were weighed and classified into five groups of five animals each, and allowed to acclimatize for two weeks. They were housed in a standard cage and maintained in standard laboratory condition at a temperature of 25±2ºC, with relative humidity of 55-64% and light and dark conditions (12/12h). They were fed with Top Feeds (Flour Mills Lagos, Nigeria.) and leaded acetate (CH_3_COO)_2_Pb·3H_2_O solution, except for the normal controls, that received deionized water *ad libitum*. Animal ethics and proper handling methods were strictly adhered to.

### Design

 Five groups of five male albino Wistar rats were used in the experiment. Each group was treated as follows for four weeks. Group 1, which served as the normal controls, received deionized water; Group 2 (toxic control) received lead acetate solution *ad libitum*. While Group 3 received lead acetate solution plus 750mg/kg bw. *Costus afer.* Group 4 received lead acetate solution plus 1500mg/kg b.w. *Costus afer* and Group 5 received lead acetate solution plus 2250mg/kg bw. *Costus afer.* The dose of the *Costus afer* extract used was based on previous studies (Ezejiofor *et al*., 2014), while the Sadeghi *et al.* method (2013) was adopted for the administration of the lead acetate solution. The body weights were monitored weekly, while the fluid and feed intake of the rats in all the groups were monitored daily for 28 days.

### Necropsy

On the 28^th^ day, the rats were fasted overnight, weighed, and slaughtered under ether anesthesia on the 29^th^ day. The blood samples were collected by cardiac puncture and kept at a temperature of 4˚C for 6 hours. The blood samples were then centrifuged at 3000 rpm for 15 minutes and stored properly for further analysis. The testis and epididymis were harvested, absolute and relative weights were measured. The blood sample was spun at 3000rpm for 10min using a centrifuge. The left testes and epididymis were stored in 10% formaldehyde and processed for histological examination, whereas the right testes and epididymis were homogenized in ice-cold 0.1M Tris HCl buffer (pH 7.4) to produce 10% homogenate. The homogenate was centrifuged at 5000g at 4^o^C for 15 minutes. The supernatant was collected and used in an antioxidant assay.

### Hormonal analyses

Plasma testosterone TET, luteinizing hormone (LH) and follicle stimulating hormone (FSH) assays were performed using a commercial microplate enzyme immunoassay kit, following the manufacturer’s instructions (Monobind Inc., USA). The testosterone AccuBind™ Microplate EIA Test System has a sensitivity of 0.0576 ng/ml and with a negligible cross reactivity with other androgen derivatives like androstenedione, 5α-dihydrotestosterone, and methyltestosterone.

### Sperm Analysis

Spermatozoa were obtained from the epididymis by the method described previously (El-Desoky *et al*., 2013). The seminal fluid was collected by macerating the epididymis in phosphate buffered saline (PBS), centrifuged at 12,000 rpm for 5 minutes and incubated at 37ºC. The supernatants were assayed for sperm quality and characteristics - as described by Cheesbrough (2006). Sperm motility was assessed by the method described by Zemjanis (1970). The motility of epididymal sperm was microscopically evaluated within 2-4 minutes of their isolation from the caudal epididymis and the data was expressed as percentages. Epididymal sperm count was obtained by mincing the caudal epididymis in distilled water and filtering through a nylon mesh. The spermatozoa were counted using the hemocytometer with the improved Neubauer (Deep 1/10m, LABART, Germany) chamber, as per described by Pant and Srivastava (Pant & Srivastava, 2003). A total of 400 spermatozoa from each rat were examined for morphological traits.

### Determination of Daily sperm production and testicular sperm number - TSN

Daily sperm production was determined using the frozen left testes from control and treated rats according to Joyce *et al.* (1993). Briefly, the testis was homogenized for 3 minutes in 25ml of physiological saline containing 0.05% (v/v) Triton X-100. Sample aliquots of 5.5µl were then placed on the hemocytometer and counted twice at 100 X magnification under the microscope to determine the average number of spermatids per sample. These values were used to obtain the total number of spermatids per testis and this number was then divided by the testes’ weights to count spermatids per gram of testes. Developing spermatids spent 4.61 days in rats. Thus 4.61 to obtain the daily sperm production (Joyce *et al.*, 1993) divided the values for the number of spermatids per testis.

### Determination of morphological abnormalities and percentage viability

The sperm suspension was placed on a glass slide, and smeared out with another slide. This was stained with Wells and Awa’s stain (0.2 g of eosin and 0.6 g of fast green dissolved in distilled water and ethanol in the 2:1 ratio) for morphological examination; and 1% eosin and 5% nigrosine in 3% sodium citrate dehydrate solution for live/dead ratio - according to the method described by Wells & Awa (1970).

### Biochemical analysis

#### Testicular/Epididymal superoxide dismutase SOD assay

We estimated the SOD using the inhibition of superoxide-dependent reduction of tetrazolium dye, methyl thiazolyl tetrazolium (MTT) to its formazan (Madesh & Balasubramanian, 1998).

#### Testicular/epididymal Malondialdehyde (MDA) Determination

Lipid peroxidation was determined by measuring the formation of thiobarbituric acid reactive substances (TBARS) (Ohkawa *et al*., 1979; Balasubramanian *et al*., 1988). The MDA level was calculated and expressed as nmol of MDA/g of wet tissue using the molar extinction coefficient of the chromophore (1.56×10^-5^/m/cm).

#### Testicular/Epididymal Reduced Glutathione (GSH) l assay

We estimated the GSH based on a reduced glutathione reaction with 5-5ditiobis-2-nitrobenzoic acid (DTNB). Testicular GSH was spectrophotometrically determined using Ellman’s reagent 5- 5-dithiobis (2-nitrobenzoic acid) (DTNB) as a chromogen at 412 nm (Sedlak & Lindsay, 1968).

#### Testicular/ Epididymal Catalase Activity assay

Catalase activity in homogenates were determined according to Clairborne (1995) with slight modifications. The specific CAT activity was calculated using the molar extinction coefficient of H_2_O_2_ at 240 nm, 43.59 l mol cm. One unit of catalase activity equals the amount of protein that converts 1 mmol H_2_O_2_ min. Activity was expressed as Units mg.

#### Testicular/Epididymal Glutathione-S-Transferase (GST) activity assay

The glutathione-S-transferase (GST) activity was determined according to Habig *et al.* (1974).

#### Testicular/Epididymal Glutathione Peroxidase Activity assay

The GSH-Px activity was assessed according to the methods described by Rotruck *et al.* (1973).

### Histopathology

Formalin fixed tissues (testes and epididymis) were dehydrated through ascending grades of alcohol, cleared in three changes of xylene, and were embedded in paraffin. Serial sections, each of 4µm thickness, were cut and stained with H and E as per standard protocols (Bancroft & Gamble, 2002). Stained sections were morphologically evaluated, and the slides were used for comparison.

### Statistical analysis

The data was analyzed using the one-way ANOVA - statistical package for social sciences (SPSS) version 12.0. The differences between mean values were tested using Duncan's multiple comparison tests and the significance level was set at *p*<0.05.

## RESULTS

### Body weight and organ weight

The weights of the animals at the experiment onset ranged from 90g in the control animals to 180 g in the Pb acetate + 2250mg/kg CA Group (Table 1). Lead acetate alone and in combination with different doses of the *C. afer* CA (750 -2250mg/kg) did not cause any significant increase in the body weight of the rats, both in control and treated Groups up to 28 days of observation (Table 1). At the end of the experiment, the percent body weight gain was high among controls (37.23%), low in the Pb-acetate group (19.87%) and even lower in decreased dosing, when compared with the controls. However, the weight increases for all groups were not significantly different after 28 days of dosing with *C. afer*. Furthermore, both the absolute and relative weights of the testes and epididymis did not change at the end of the experiment (Table 1).

**Table 1:** Effect of the aqueous leave extract of Costus afer (CA) on the body weight, absolute and relative weights of organs

Treatment Groups	Final Body weight	Organs	Absolute weight (g)	Relative weight (%)
	(weight gain/% weight gain)			
Water	99.9±7.77	Epididymis	0.27±0.01	0.27
	(27.10±7.45/37.23)	Testes	1.20±0.06	1.20
Pb Alone	114.52±11.3	Epididymis	0.60±0.00	0.52
	(18.98±6.43/19.87)	Testes	1.33±0.05	1.20
Pb+750mg/kg CA	125.7±10.2	Epididymis	0.13±0.00	0.10
	(21.04±6.65/22.46)	Testes	1.40±0.06	1.10
Pb+1500mg/kg CA	130±9.34	Epididymis	0.17±0.01	0.13
	(20.8±6.23/19.05)	Testes	1.47±0.02	1.10
Pb+2250 mg/kg CA	196.3±11.6	Epididymis	0.11±0.00	0.08
	(28.98±12.39/17.22)	Testes	1.57±0.04	0.80

Data are presented as the mean±SD (n=5). There was no significant difference among the treatment groups and control values (*p*>0.05).

### Epididymal and testicular Sperm characteristics

The effect of *C. afer* on the sperm characteristics (volume, pH, viability, morphology and epididymal sperm number) of the lead-exposed male rats are shown on Table 2. The volume, sperm motility and epididymal sperm count in the lead acetate Group alone decreased significantly (*p*<0.05), while the percent abnormal, sluggishness and dead sperm cells increased significantly (*p*<0.05) when compared with the Control Group. The sperm motility, volume, percent viability and epididymal sperm number of the *C. afer* Group were not significantly different from the control values as seen in Table 2.

**Table 2:** Effect of *C. afer* extract on the Sperm Characteristics of Lead exposed male rats

Treatment/parameter	Vol (ml)	pH	Viability (%)	Normal Morph (%)	Abnormal (%)	Motility (%)	Sluggish (%)	Dead (%)	ESN ml (*10^6^)
Water									
Mean±SD	0.12±0.07	8.0 ±0.0	71.7±10.4	65.0 ±13.2	26.6±10.4	63.3±23.6	5.33±2.9	25±18.0	3.9±2.0
(Max-Min)	0.2-0.05	8.0-8.0	80-60	75-50	35-15	90-45	10-5	40-5	5.0-1.0
Pb alone									
Mean±SD	0.08±0.03	8.0±0.0	65.0±5.0	60.0±5.0*	40.0±5.4*	45.0±5.0*	15.0±5.0*	43.3±5.9*	3.0±1.0
Max-Min	0.2-0.1	8.0-8.0	65-55	65-55	45-35	50-40	20-10	50-40	4.0-2.0
Pb+750mg/kg CA									
Mean±SD	0.13±0.06	8.0±0.0	65.0±13.2	63.±7.64**	35.0±13.2**	58.3±2.9**	11.67±2.9*	30±0	4.2±1.4**
Max-Min	0.2-0.1	8.0-8.0	75-50	70-55	50-25	60-55	15-10	30-30	5.0-2.5
Pb+1500mg/kg CA									
Mean±SD	0.13±0.06	8.0±0.0	68.3±7.63**	63.3±10.4**	36.7±10.4**	61.7±16.7**	11.67±5.8**	23.3±11.5**	4.3±7.6**
Max-Min	0.2-0.1	8.0-8.0	75-60	75-55	45-25	80-50	15-5	30-10	2.0-5.0
Pb+2250mg/kgCA									
Mean±SD	0.17±0.0	8.0±0.0	78.3±10.4**	71.67±12.6**	35.0±8.67**	78.3±2.89**	11.67±2.9**	13.3±2.8**	4.6±1.0**
Max-Min	0.1-0.05	8.0-8.0	90-70	70-55	45-30	80-75	15-10	15-10	6.0-4.0

Data expressed as mean±S.D.

* Values differ significantly from control (*p*<0.05).

** Values differ significantly from Pb alone (*p*<0.05). CA = *C. afer*

#### Hormonal parameters

**Figure 1:**
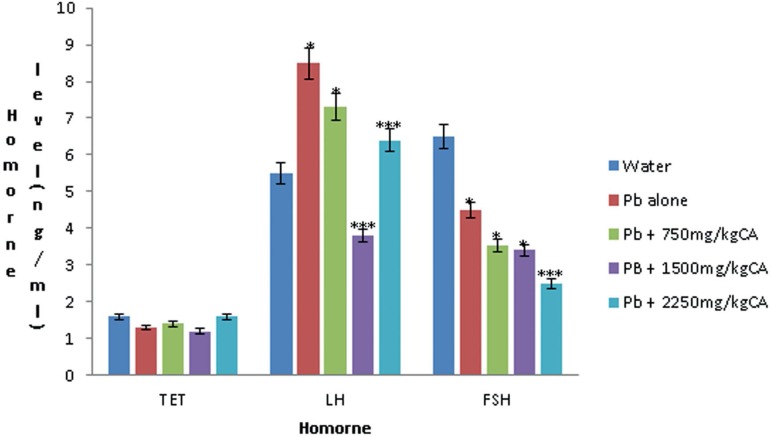
Effect of *C. afer* extract on the hormonal (ng/ml) parameters of lead-exposed rats. n=5, *(significantly different from the water); ***(significantly different from the lead alone).

The effect of *C. afer* on the plasma testosterone (TET), Luteinizing hormone (LH) and Follicle stimulating hormone (FSH) levels on the lead-exposed male rats is shown in Figure 1. There was no significant difference in the TET level in lead acetate only (1.3±0.00) and lead acetate plus *C. afer*-treated Groups (1.4±0.1, 1.2±0.06, 1.6±0.1). Whereas a significant difference (*p*<0.05) was seen in the LH and FSH levels in lead acetate only (LH-8.5±1.4: FSH-4.5±2.6) and in the lead acetate plus *C. afer* treated groups (LH-7.3±0.5, 3.8±0.6, 6.4±0.5 and FSH-3.53±0.15, 3.2±1.1, 2.5±0.17 compared with the normal control animals (LH- 5.5±0.4 and FSH-6.5±1.65).

#### Testicular MDA and GSH level, GSH-Px and GST activities

**Figure 2:**
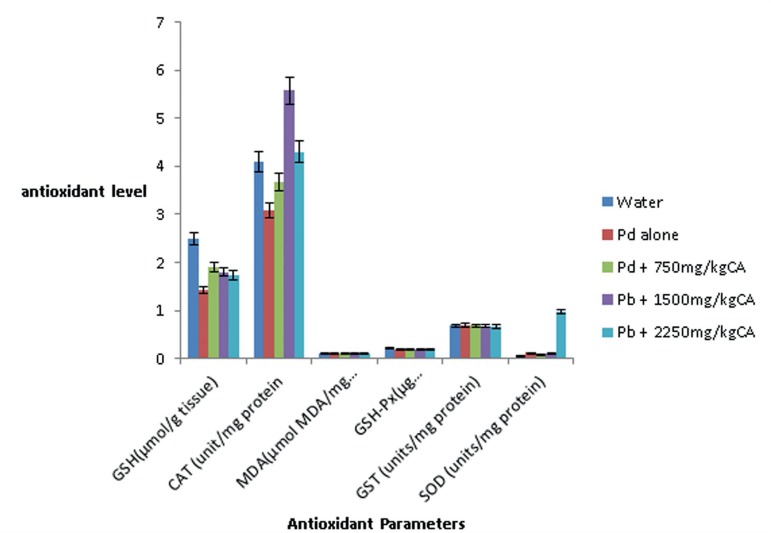
Effect of aqueous leave extract of *C. afer* on the testicular anti-oxidant parameters in lead-exposed rats

Figure 2 shows the changes in MDA and GSH levels, as well as GST and GSH-Px activities in the testes. There were no significant changes in the level of testicular MDA, GST and GSH-Px activity in the testes of Pb acetate alone treated rats compared to those in the Control Group (0.11± 0.01mol MDA mg *versus* 0.11± 0.01mol MDA mg^-1^, 0.69±0.03 *versus* 0.68±0.03 and 0.22±0.04 *versus* 0.18±0.03g residual GSH remaining min mg respectively). There were no significant changes in the testicular MDA level, GST and GSH-Px activities in the lead acetate + *C. afer* treated groups compared to control animals (*p*>0.05).

#### Changes in testicular antioxidant enzymes (CAT and SOD) activities

There were 25% and 10% decrease in testicular CAT activities in the Pb acetate alone and 750mg/kg CA treated rats, respectively, when compared with the Control Group (Figure 2); whereas there were 40% and 7.6% increases in lead acetate plus 1500mg/kg CA and lead acetate plus 2250mg/kg CA treated groups, respectively. The difference between the CAT activity in the lead acetate plus 1500mg/kg CA treated and control animals was statistically significant (*p*<0.05). The testicular CAT activity increased 1.36 fold (4.1±0.46 *versus* 5.57±0.51unit mg) in the Pb acetate plus 1500mg/kg CA rats, compared to Pb acetate only treated rats (Figure 2). There was no significant change in the testicular SOD both in the Pb acetate only and *C. afer* extract treated animals (Figure 2).

#### Epididymal MDA and GSH level, GSH-Px and GST enzyme activities

**Figure 3:**
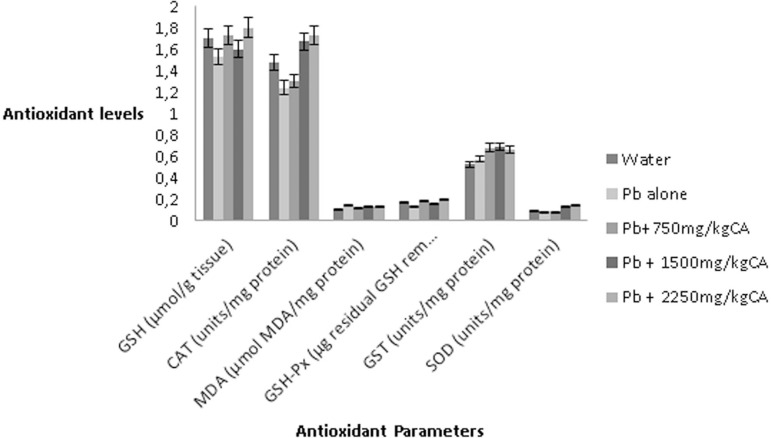
Effect of aqueous leave extract of *C. afer* on the epididymal anti-oxidant parameters in lead-exposed rats

The effect of *C. afer* on the MDA and GSH levels, as well as GST and GSH-Px activities in epididymis are summarized in Figure 3 on lead exposed male rats. There was no significant change in the level of epididymal MDA, GST and GSH-Px activity in the epididymis of Pb acetate only treated rats compared to control values. There were also no statistical significant changes in epididymal MDA level, GST and GSH-Px activity in lead acetate plus *C. afer*-treated rats compared to controls (*p*>0.05).

#### Changes in epididymal antioxidant enzymes (CAT and SOD) activities

The epididymal CAT and SOD showed no changes in both the Pb acetate only and lead acetate plus *C. afer*-treated rats (Figure 3) when compared with the Control Group.

#### Daily Sperm Production (DSP), Testicular Sperm Number (TSN) and Blood Lead Level (BLL).

**Figure 4:**
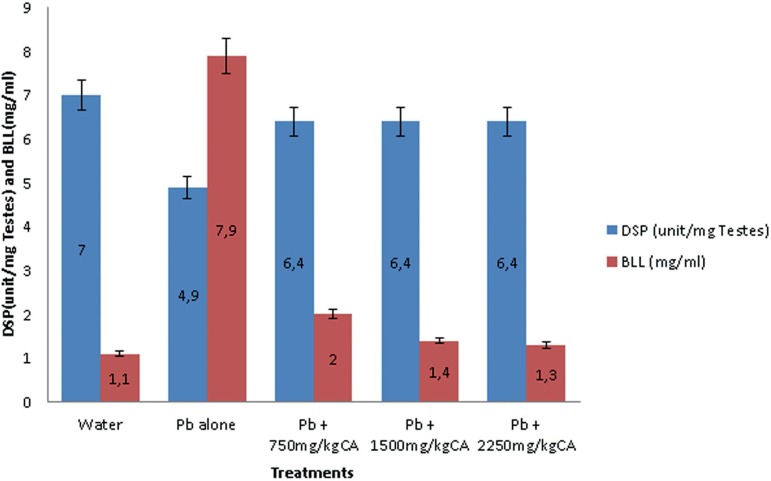
Effects of *C. afer* on Daily Sperm Production (DSP) and Blood Lead Level (BLL) in lead-exposed rats. Data is expressed as mean±S.D.; n=5

**Figure 5:**
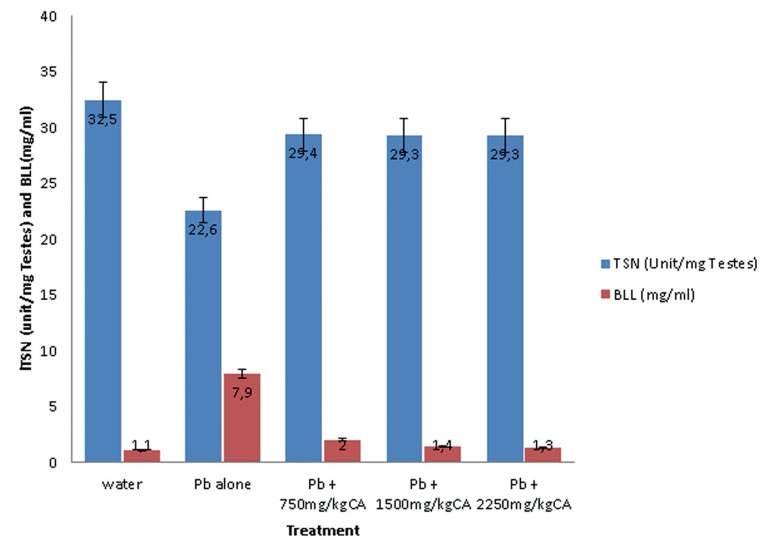
Effects of *C. afer* on testicular sperm number (TSN) and Blood Lead Level (BLL) in lead exposed rats. Data is expressed as mean±S.D.; n=5

The effects of *C. afer* on daily sperm production (DSP) and Blood Lead Level (BLL) and testicular sperm number (TSN) and Blood Lead Level (BLL) in lead acetate treated male albino rats are shown in Figure 4 and 5, respectively. The DSP decreased and BLL increased in Pb acetate animals compared with the control. There was significant increase in DSP and a decrease in BLL following treatment with the *C. afer* CA extract.

A similar trend was seen in Figure 5, with a decrease in Testicular Sperm Number TSN and an increase in BLL in the Pb acetate animals compared with the control value. In the *C. afer* treated animals, the reverse was the case with an increase in TSN and a decrease in BLL, respectively.

### Histological examination of the testis

**Figure 6:**
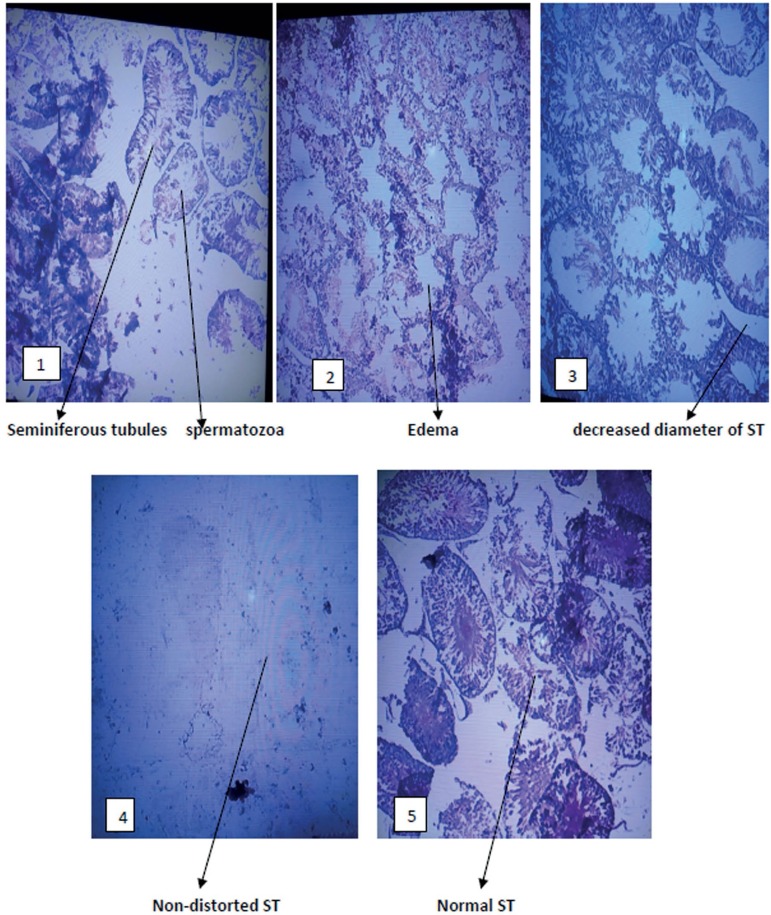
Photomicrograph of the testes: 1 (H_2_O), 2 (Pb alone), 3(Pb+750mg/kg CA), 4 (Pb+1500mg/kg CA) and 5 (Pb+2250mg/kg CA). All panels were stained with hematoxylin & eosin. Magnification x100. ST (Seminiferous tubules). CA=*Costus afer*

**Figure 7:**
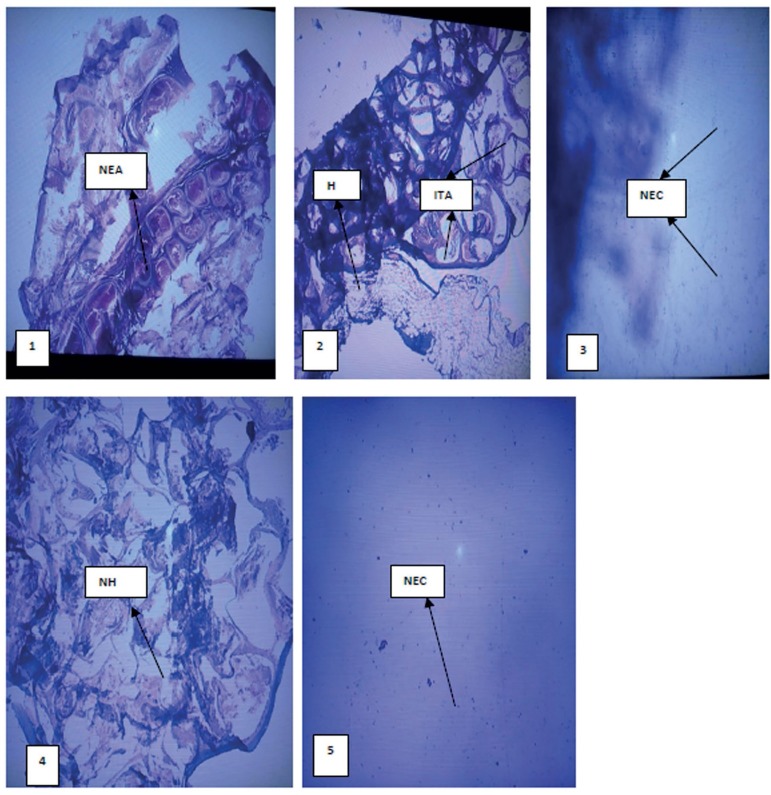
Photomicrograph of the epididymis: 1 (H_2_O), 2 (Pb alone), 3(Pb+750mg/kg CA), 4 (Pb+1500mg/kg CA) and 5 (Pb+2250mg/kg CA). All panels were stained with hematoxylin & eosin, magnification x100. NEC (normal epididymal cell), NEA (normal epididymal architecture), ITA (inflamed tunica albuginea), H (hydrocele)

Figures 6 and 7 show the histopathology of the testes treated with lead acetate and *C. afer*. There were no histopathological changes in the testes and epididymis of rats treated with *C. afer* when compared with lead acetate only treated group. Edema, hydrocele and inflamed tunica albuginea were observed in the lead acetate only treated group. Such effect was alleviated by *C. afer*

## DISCUSSION

The testicular toxicity of Pb is mediated by oxidative damage and generation of reactive oxygen species (ROS) (Morán-Martínez *et al*., 2013; Fahim *et al*., 2013). The pathological role of ROS in infertility has been studied but not well established due to the various possible sources associated with excess production of ROS, including abnormal spermatozoa (Venkatesh *et al*., 2009). Oxidants seem to interfere with normal sperm function via peroxidation of unsaturated fatty acids in the sperm plasma membrane, which results in sperm dysfunction (Barros *et al*., 2003). Mammalian spermatozoa are coated with a membrane rich in polyunsaturated fatty acids (PUFA) which are very susceptible to oxidative damage by free radicals or ROS. The lipid peroxidation (LPO) mechanism damages the sperm cell membrane and is thought to be the main feature of the ROS-induced sperm damage leading to loss of motility, abnormal morphology and reduced capacity for sperm oocyte penetration and infertility (Storey, 1997). The body depends on strong antioxidant enzymes, such as superoxide dismutase (SOD), catalase, and glutathione peroxidase/reductase for its proper function. Storey (1997) reported that glutathione peroxidase/reductase enzymes play a central role in the defense against oxidative damage in human sperm. Seminal plasma and spermatozoa have abundance of antioxidant enzymes, namely glutathione peroxidase, glutathione reductase, superoxide dismutase (Yeung *et al*., 1998), and some of these antioxidant enzymes are made by the epididymis during storage (Potts *et al*., 2000). A decrease in the levels of reduced glutathione (GSH) during sperm production disrupts the membrane integrity of spermatozoa because of increased oxidative stress. GSH peroxidase, a selenium-containing antioxidant enzyme with GSH as the electron donor, removes peroxyl radicals from various peroxides including H_2_O_2_. GSH reductase then regenerates reduced GSH from oxidized GSH (GSSG). A selenium-associated polypeptide, presumably GSH peroxidase, has been demonstrated in rat sperm mitochondria; it plays a significant role in this peroxyl scavenging mechanism and, ultimately, in maintaining sperm motility. Although no significant changes were seen in the epididymal antioxidant parameters, treatment with *C. afer* significantly increased the levels of testicular GSH, CAT and SOD when compared with the Pb-acetate-only group. These antioxidant properties of *C. afer* may be responsible for it protective effect against the Pb-acetate-induced testicular toxicity (Dorostghoal *et al*., 2014; Sharma & Garu, 2011; Ansar *et al*., 2016). Pb can cross the blood-testis barrier, build up in the testis, and damage germinal cells at various levels of differentiation, shifting the oxidant/antioxidant profile towards the oxidant side as manifested by the marked exhaustion of the enzymatic antioxidants together with the buildup of lipid peroxidation products in the testicular tissue homogenate.

Administration of *C. afer* in the Pb-acetate-treated rats seemed to significantly restore seminal volume, pH, viability, morphology and ESN. There are reports of certain reductions in testis volume, seminiferous tubules diameter and germinal epithelium height increase from early weeks to 60 days of age, nearly by the onset of puberty, but it decreases afterward, so it seems that Pb has transient effects and testicular parameters become gradually better until 120 days of age. A plausible mechanism in Pb-toxicity is the loss of tissue homeostasis via an imbalance between pro and anti-oxidative factors (El-Masry *et al*., 2016). In a dose-dependent fashion, Pb tends to enter the tight junctions of the inter-Sertoli barrier, damage the epithelium, with a decrease in its height due to germ cell loss, thus enlarging the tubular lumen. *Costus afer* may hold a promise in the management of Pb-induced testicular toxicity as evidenced by it restorative effect on the various seminal parameters.

The major function of the testes is spermatogenesis and hormone production (Brennan & Capel, 2004), hence the testicular toxicity of Pb ultimately causing reduction in male sex hormones (Chowdhury, 2009). Besides the production of spermatozoa, testes are involved in the production of hormones that are required for various functions in the body, including maintenance of secondary sexual functions, and feedback on the hypothalamus and the pituitary to control the secretion of the gonadotropins. TET, LH and FSH are important hormonal components of male sexual development and fertility. A significant decline in TET or an increase in LH and FSH has been shown to adversely affect sexual maturity and fertility in male animals (Mann & Lutwak-Mann, 1981). In the present study, there was a significant increase in LH and FSH and a decrease in TET, found in the toxic control, which differs significantly from the normal controls, confirming the previous findings of Mann and Lukwat-Mann (Mann & Lutwak-Mann, 1981). It could be inferred that *C. afer* confers a protective effect by bringing to near normal the level of plasma TET, LH and FSH in the treated groups.

The blood lead level (BLL) was inversely related to the sperm production (DSP) and testicular sperm number (TSN). *C. afer* administration significantly increased both the DSP and TSN with accompanying reductions in BLL. Several studies in many rat strains and rodents indicate fairly consistently that blood lead (Pb) concentration > 30-40 µg/dl during at least 30 days of administration was associated with spermatogenesis impairment and reduced concentration of circulating androgens (Pandya *et al*., 2012; Assi *et al*., 2016). In the present study, there were increases in BLL coupled with daily decreases in sperm production (DSP), testicular sperm number (TSN) and reduced fertility indices (sperm concentration, percentage viability, individual motility and general motility) and increased percentage abnormality following lead acetate administration. These effects were either significantly reversed or brought to near normal control levels after treatment with *C. afer.* Histological analysis of the testis and epididymis showed marked distortions in the Pb-acetate-treated group compared with controls and *C. afer-*treated groups.

## CONCLUSION

The present study implicated Pb as a reprotoxicant, affecting both the histological, biochemical and sperm analyses of the exposed rats, in general causing overall reproductive damage which was ameliorated by *Costus afer*. Taken together, aqueous leaf extract of *C. afer* may hold promise in alleviating Pb-induced male reproductive damage.
